# A Multielement Prognostic Nomogram Based on a Peripheral Blood Test, Conventional MRI and Clinical Factors for Glioblastoma

**DOI:** 10.3389/fneur.2022.822735

**Published:** 2022-02-09

**Authors:** Changjun Rao, Jinghao Jin, Jianglong Lu, Chengde Wang, Zerui Wu, Zhangzhang Zhu, Ming Tu, Zhipeng Su, Qun Li

**Affiliations:** Department of Neurosurgery, The First Affiliated Hospital of Wenzhou Medical University, Zhejiang, China

**Keywords:** glioblastoma, prognosis, nomogram, VEGFA, survival

## Abstract

**Background:**

Glioblastoma (GBM) is one of the most malignant types of tumors in the central nervous system, and the 5-year survival remains low. Several studies have shown that preoperative peripheral blood tests and preoperative conventional Magnetic Resonance Imaging (MRI) examinations affect the prognosis of GBM patients. Therefore, it is necessary to construct a risk score based on a preoperative peripheral blood test and conventional MRI and develop a multielement prognostic nomogram for GBM.

**Methods:**

This study retrospectively analyzed 131 GBM patients. Determination of the association between peripheral blood test variables and conventional MRI variables and prognosis was performed by univariate Cox regression. The nomogram model, which was internally validated using a cohort of 56 GBM patients, was constructed by multivariate Cox regression. RNA sequencing data from Gene Expression Omnibus (GEO) and Chinese Glioma Genome Atlas (CGGA datasets were used to determine peripheral blood test-related genes based on GBM prognosis.

**Results:**

The constructed risk score included the neutrophil/lymphocyte ratio (NLR), lymphocyte/monocyte ratio (LMR), albumin/fibrinogen (AFR), platelet/lymphocyte ratio (PLR), and center point–to-ventricle distance (CPVD). A final nomogram was developed using factors associated with prognosis, including age, sex, the extent of tumor resection, IDH mutation status, radiotherapy status, chemotherapy status, and risk. The Area Under Curve (AUC) values of the receiver operating characteristic curve (ROC) curve were 0.876 (12-month ROC), 0.834 (24-month ROC) and 0.803 (36-month ROC) in the training set and 0.906 (12-month ROC), 0.800 (18-month ROC) and 0.776 (24-month ROC) in the validation set. In addition, vascular endothelial growth factor A (VEGFA) was closely associated with NLR and LMR and identified as the most central negative gene related to the immune microenvironment and influencing immune activities.

**Conclusion:**

The risk score was established as an independent predictor of GBM prognosis, and the nomogram model exhibit appropriate predictive power. In addition, VEGFA is the key peripheral blood test-related gene that is significantly associated with poor prognosis.

## Background

Glioblastomas (GBMs) are prevalent malignant tumors of the brain and the central nervous system (CNS), causing most deaths among primary brain tumor patients (1). Despite advances in cancer diagnosis and treatment, no significant improvements in patient outcomes have been reported. GBM patients have poor clinical outcomes with 5-year relative survival rates and median overall survival times of 5.6% and 12–15 months, respectively (2, 3). Therefore, it is necessary to determine appropriate prognostic factors for suitable risk classification. Studies have found several molecular markers, including the methylation state of the O6-methylguanine-DNA methyltransferase (MGMT) gene promoter and isocitrate dehydrogenase enzyme 1/2 (IDH1/2) mutation. However, these markers are not preoperatively available. Therefore, there is a need to develop a reliable and simple preoperative scoring system for the prediction of GBM prognosis (4–9).

Peripheral blood tests can significantly impact tumor growth (10). Peripheral blood tests, such as NLR, PLR, LMR, red cell volume distribution width (RDW), albumin/globulin ratio (AGR), albumin/gamma-glutamyl transferase ratio (AGgR) and fibrinogen, can refine patient stratification to therapy and predict survival outcomes in gliomas (11–14). Moreover, conventional MRI offers diagnostic value for genotype classification (15). We aimed to build a risk score based on peripheral blood tests and conventional MRI for GBM prognosis. A nomogram was also developed to predict individualized survival outcomes for GBM patients using this scoring system, age, sex, IDH mutation, and other important prognostic factors. RNA sequencing data from the Chinese Glioma Genome Atlas (CGGA) were used to determine key peripheral blood test-related genes based on GBM prognosis.

## Materials and Methods

### Study Population

This study retrospectively reviewed 233 patients pathologically diagnosed with WHO grade IV GBM at the First Affiliated Hospital of Wenzhou Medical University from March 2011 to March 2020. The inclusion criteria for this study were as follows: (1) Age ≥ 18 years. (2) Patients with first-time preoperative peripheral blood cell data for GBM samples. (3) Patients without acute conditions, such as microbial infections, or those patients who underwent drug treatments that could have affected the immune system. (4) Patients with complete clinical data as well as follow-up data. A total of 187 patients met our inclusion criteria and were included in our study. A total of 131 patients treated from March 2011 to September 2018 were included in the training set, whereas 56 patients identified from October 2018 to March 2020 were included in the validation set. This study followed the Declaration of Helsinki and was permitted by the review board of the First Affiliated Hospital of WMU. Patients were required to sign informed consent for the use of their clinical data in future studies.

RNA expression profiles of immune cell subsets of humans were retrieved from Gene GEO (https://www.ncbi.nlm.nih.gov/geo) datasets (GSE28491). Molecular pathology and RNA sequencing data as well as clinical information for 693 glioma patients were retrieved from the CGGA database (http://www.cgga.org.cn), and we also retrieved RNA sequencing data of GBM from The Cancer Genome Atlas (TCGA; http://tcga-data.nci.nih.gov/tcga/), GSE16011 (16). RNA sequencing data of normal tissues were acquired from the Genotype-Tissue Expression Project (GTEx) (http://www.gtexportal.org) and log2(X+1) transformed for subsequent analyses. Single-cell sequencing (ScRNA-seq) data of key genes were obtained from Cancer Single-cell State Atlas (CancerSEA) (http://biocc.hrbmu.edu.cn/CancerSEA/), Patel AP. Science. 2014 (Brain) (EXP0058) (GSE57872) (17).

### Data Collection

The patients' clinical parameters, including sex, tumor location, age, tumor size, resection extent, postoperative adjuvant chemoradiotherapy, preoperative fibrinogen, preoperative neutrophil and lymphocyte counts, preoperative albumin and globulin levels, and preoperative gamma-glutamyl transferase, were collected from the electronic medical records system.

Two blinded neuroradiologists with ≥7 years of MR as well as CT neuroimaging experience independently reviewed MRI scans. The assessed imaging features were (1) ring-shaped gadolinium enhancement (RE) (18) (presence or absence), (2) peritumoural oedema (presence or absence), (3) involvement of the subventricular zone (SVZ) ion on T2WI (presence or absence), (4) involvement of the insula on T2WI (presence or absence), (5) center point–to-ventricle distance (CPVD), and (6) tumor volume (D1^*^D2^*^D3/2) (19– 21).

### Statistical Analysis

A log-rank test was used to establish the optimal threshold values for NLR, LMR, PLR, CPVD, and AFR. Continuous variables are expressed as numbers and means ± SD and were compared with unpaired Student's *t*-tests. Categorical data were assessed using the chi-square test. Correlations were calculated using Spearman's rank correlation. Kaplan–Meier survival curves were used for survival analysis (22–24). Cox proportional hazards regression models were used for univariate and multivariate analyses of clinical variables for the determination of independent prognostic factors. Finally, differentially expressed genes were examined using the Wilcoxon test. Univariate Cox regression, LASSO regression and multivariate Cox regression were performed to select the key genes. *p* ≤ 0.05 denoted significance, and all *p* values were two-sided. SPSS 22.0 was used for all statistical analyses.

### Establishment and Validation of the Nomogram

The risk score system was calculated as follows: GBM patients with high NLR (>4.16), high PLR (>193.75), low LMR (≤2.52), low AFR (≤9.43), and short CPVD (≤32.56) had a score of 5 (five abnormalities). A risk score of 0 was defined as low risk; risk scores 1 and 2 were defined as moderate risk; risk scores 3 and 4 were defined as high risk; and a risk score of 5 was defined as ultra-high risk. With the exception of the number of chemotherapies in the training set, the four risk groups did not differ in participant characteristics at baseline in either the training set or validation set. Then, independent prognostic factors, such as age, sex, extent of resection (EOR), IDH mutation, postoperative adjuvant chemoradiotherapy and risk score, were screened by multivariate Cox regression analysis and used to construct a nomogram to predict the 12-, 24-, and 36-month overall survival (OS) outcomes of GBM. A “nomogramEx” package was used to obtain each patient's point in the nomogram. Receiver operating characteristic (ROC) curves were drawn, and the area under the curve (AUC) was determined to test the predictive significance of the nomogram. Moreover, the nomogram calibration curve was plotted to approximate the predictive ability of the nomogram. The models were trained and validated using the training and validation set data, respectively.

### Exploration of Peripheral Blood Test-Related Genes

The downloaded RNA expression profiles of human immune cell subsets (GSE28491, 5 neutrophil samples, 5 monocyte samples and 20 lymphocyte samples) were used to explore the peripheral blood test-related genes that also influence the immune infiltrates of GBM. The edgeR package in R was used for the analysis of differential expression between neutrophils and lymphocytes as well as monocytes and lymphocytes. Genes with *p* < 0.05 were determined to be differentially expressed genes. Signatures related to NLR and LMR were denoted as significantly differentially expressed genes between neutrophils and lymphocytes and between monocytes and lymphocytes. The differentially expressed genes were also screened between CGGA and GTEx, representing normal and GBM tissues. Finally, various immune-associated genes were obtained from ImmPort (www.immport.org/shared/genelists). Thirty-eight genes were expressed in the final intersection and were used for the subsequent analysis. Genes that were markedly associated with prognosis were assessed by univariate Cox regression analysis at a cut-off *p* value < 0.05. The training set was further subjected to LASSO regression analysis and multivariate Cox regression analysis to identify the best prognostic genes via the glmnet package in R (22). Based on GBM prognosis, the key genes (SLC11A1 and VEGF) were selected by multivariable Cox regression. Therefore, VEGFA was determined to be a key prognostic-associated immune-related gene (IRG) by combining univariate Cox regression and PPI network analyses with clinical factors. Finally, RNA sequencing data from CGGA, TCGA and GSE16011 were used to calculate the correlation between NLR, LMR and expression of VEGFA. This was achieved with the CIBERSORT algorithm based on the deconvolution, using the ‘CIBERSORT' R package. The thresholds for inclusion was *P* ≤ 0.05 (25).

### Multivariate Analysis of VEGFA

Correlations between VEGFA expression and clinical factors, including sample type, gender, age, IDH mutation status,1p19q codeletion status and MGMT promoter methylation status were evaluated. GO and KEGG analyses of VEGFA were performed via gene set enrichment analysis (GSEA) (26). Single-sample gene set enrichment analyses (ssGSEA) were performed using “gsva” in R. Infiltration scores for 16 immune cells as well as the activities of 13 immune-associated pathways were determined for each sample in the 693 CGGA dataset. Subsequently, the correlation among the expression level of the key genes, immune-related pathways, immune cell infiltration and special immune checkpoints in GBM were assessed. Finally, the relationship between VEGFA and gene functional states were analyzed by CancerSEA (17).

## Results

### Patient Characteristics

Patient demographics for the imputed, training set (*N* = 131), and validation set (*N* = 56) are shown in Table 1. Patients in the training set were significantly younger than those in the validation set (*p* = 0.016). Most patients in the training set harbored IDH mutations, whereas most patients in the validation set had gross total resection. Compared to the training set, a higher number of patients in the validation set received current radiation and TMZ. Finally, compared to the validation set, more patients died in the training set (*P* < 0.001).

**Table 1:** Characteristics of GBM patients in the training and validation sets.

	**Training set (*N* = 131)**	**Validation set (*N* = 56)**	***P* value**
Age [mean (SD)] [Range]	52.53 (14.21) [18–79]	57.95 (13.35) [22–79]	0.016
Gender [*N* (%)]			0.598
Male	71 (54.2)	28 (50)	
Female	60 (45.8)	28 (50)	
IDH-Mutation [*N* (%)]			0.155
Mutant	16 (12.2)	3 (5.4)	
Wildtype	115 (87.8)	53 (94.6)	
EOR [*N* (%)]			0.292
Total	101 (77.1)	47 (83.9)	
Not total	30 (22.9)	9 (16.1)	
Radiotherapy [*N* (%)]			0.858
Treated	73 (55.7)	32 (57.1)	
Untreated	58 (44.3)	24 (42.9)	
Chemotherapy (TMZ) [*N* (%)]			0.246
Treated	100 (76.3)	47 (83.9)	
Untreated	31 (23.7)	9 (16.1)	
Survival status			<0.001
Alive	16 (12.2)	20 (35.7)	
Dead	115 (87.8)	36 (64.3)	

*EOR, extent of resection; TMZ, temozolomide*.

### Calculation of the Scoring System

The point with the minimum *P* value of the log-rank test was considered to be the optimal cut-off value. The cut-off values were 4.16, 2.52, 193.75, 9.43, 1.61 and 2.61 for NLR, LMR, PLR, AFR, AGR and AGgR, respectively. The AGR and AGgR values were removed given the lack of significant results in the KM survival curve. In addition, the differences in overall survival caused by the presence or absence of ring-shaped gadolinium enhancement (RE), peritumoural and subventricular zone (SVZ) involvement on T2WI and insular involvement on T2WI on MRI were not statistically significant. The cut-off values were 32.56 mm and 48.44 cm^3^ for CPVD and the tumor volume, respectively. However, no statistically significant results for volume were obtained. Therefore, the scoring system contained the following predictor variables: NLR, PLR, LMR, AFR, and CPVD. The risk score was defined as follows: patients with high NLR (>4.16), high PLR (>193.75), low LMR (≤2.52), low AFR (≤9.43), and short CPVD (≤32.56) received a score of 5 (five abnormalities). Each abnormality was assigned one point. If all 5 parameters did not meet the standards, the patient was given a score of 0 (no abnormality). Approximately 18.3, 29.0, 28.2, 13.7, 8.4, and 2.3% of the patients had risk scores of 0, 1, 2, 3, 4, and 5, respectively. The median overall patient survival was 23.6 months, 15.4 months, 12.6 months, 6.1 months, 6.6 months and 6.0 months for risk scores of 0, 1, 2, 3, 4 and 5, respectively (*P* < 0.001) (Table 2).

**Table 2:** The cut-off values for NLR, PLR, LMR, AFR, AGR, AGgR, CPVD and volume.

**Variables**	**No. (%)**	**mOS [months (95% CI)]**	***P value***
NLR			0.009
>4.16	51 (38.9)	7.3 (4.8–9.8)	
≤4.16	80 (61.1)	16.8 (14.4–19.2)	
PLR			0.040
>193.75	31 (23.7)	10.5 (3.5–17.5)	
≤193.75	100 (76.3)	15.4 (12.4–18.4)	
LMR			0.021
>2.52	92 (70.2)	15.5 (12.9–18.1)	
≤2.52	39 (29.8)	9.3 (1.2–17.4)	
AFR			0.001
>9.43	108 (82.4)	15.8 (12.7–18.9)	
≤9.43	23 (17.6)	10.5 (5.8–15.2)	
AGR			0.186
>1.61	18 (13.7)	15.8 (0.0–33.5)	
≤1.61	113 (86.3)	14.0 (11.0–17.1)	
AGgR			0.304
>2.61	26 (19.8)	18.3 (8.2–28.4)	
≤2.61	105 (80.2)	13.6 (10.9–16.4)	
CPVD			0.045
>32.56 mm	50 (38.2)	17.2 (15.2–19.2)	
≤32.56 mm	81 (61.8)	11.7 (7.3–16.1)	
Volume			0.224
>48.44 cm^3^	33 (25.2)	18.1 (13.3–22.9)	
≤48.44 cm^3^	98 (74.8)	13.0 (9.6–16.4)	
SVZ			0.488
Yes	63 (48.1)	13.2 (8.0–18.5)	
No	68 (51.9)	16.0 (13.4–18.6)	
RE			0.265
Yes	60 (45.8)	16.8 (14.4–19.2)	
No	71 (54.2)	13.6 (11.0–16.2)	
Insular			0.151
Yes	60 (45.8)	12.6 (8.8–16.4)	
No	71 (54.2)	17.2 (13.8–20.6)	
Edema			0.383
Yes	117 (89.3)	14.0 (5.5–24.5)	
No	14 (10.7)	15.0 (11.7–16.3)	
Risk score			<0.001
0	24 (18.3)	23.6 (8.5–38.7)	
1	38 (29.0)	15.4 (12.5–18.3)	
2	37 (28.2)	12.6 (6.0–19.2)	
3	18 (13.7)	6.1 (0.0–22.5)	
4	11 (8.4)	6.6 (3.6–9.6)	
5	3 (2.3)	6.0 (0.0–13.2)	

*Median overall survival outcomes of the prognostic factors*.

### Univariate and Multivariate Analyses for OS

These analyses were conducted using clinical data of the training set. Univariate analysis revealed that age (hazard ratio (HR) = 1.034; 95% CI = 1.020–1.049; *P* < 0.001), extent of resection (EOR) (HR = 0.374; 95% CI = 1.060–2.704; *P* = 0.027), IDH mutation (HR = 2.848; 95% CI = 1.438–5.643; *P* = 0.003), radiotherapy (HR = 3.139; 95% CI = 2.138–4.609; *P* < 0.001), chemotherapy (HR = 4.531; 95% CI = 2.892–7.100; *P* < 0.001) and risk score (*P* < 0.001) were markedly correlated with OS. Furthermore, multivariate analysis showed that age (HR = 1.030; 95% CI = 1.013–1.047; *P* < 0.001), EOR (HR = 0.543; 95% CI = 0.321–0.919; *P* = 0.023), IDH mutation (HR = 2.275; 95% CI = 1.090–4.749; *P* = 0.029), radiotherapy (HR = 2.072; 95% CI = 1.269–3.385; *P* = 0.014), chemotherapy (HR = 2.044; 95% CI = 1.142–3.660; *P* = 0.006) and risk score (*P* = 0.002) were independent predictive factors for OS (Table 3).

**Table 3:** Univariate and multivariate Cox analyses of OS.

**Variables**	**Univariate analysis**		**Multivariate analysis**	
	**HR (95% CI)**	***P* value**	**HR (95% CI)**	***P* value**
Age	1.034 (1.020–1.049)	<0.001	1.030 (1.013–1.047)	<0.001
Gender (male/female)	0.940 (0.652–1.357)	0.940	0.827 (0.557–1.228)	0.347
EOR (total/not total)	0.374 (0.240–0.581)	<0.001	0.543 (0.321–0.919)	0.023
IDH (wildtype/mutant)	2.848 (1.438–5.643)	0.003	2.275 (1.090–4.749)	0.029
Radiotherapy (untreated/treated)	3.139 (2.138–4.609)	<0.001	2.072 (1.269–3.385)	0.004
Chemotherapy (untreated/treated)	4.531 (2.892–7.100)	<0.001	2.044 (1.142–3.660)	0.016
Risk score		<0.001		0.002
Risk score 0	1 (reference)		1 (reference)	
Risk score 1	1.190 (0.667–2.124)	0.557	1.433 (0.789–2.603)	0.238
Risk score 2	1.954 (1.114–3.428)	0.019	1.437 (0.809–2.553)	0.216
Risk score 3	2.095 (1.087–4.037)	0.027	2.484 (1.262–4.891)	0.008
Risk score 4	3.688 (1.728–7.872)	0.001	3.742 (1.692–8.280)	0.001
Risk score 5	8.492 (2.411–29.903)	0.001	8.134 (2.144–30.862)	0.002

### Nomogram and Validation

**Figure 1:**
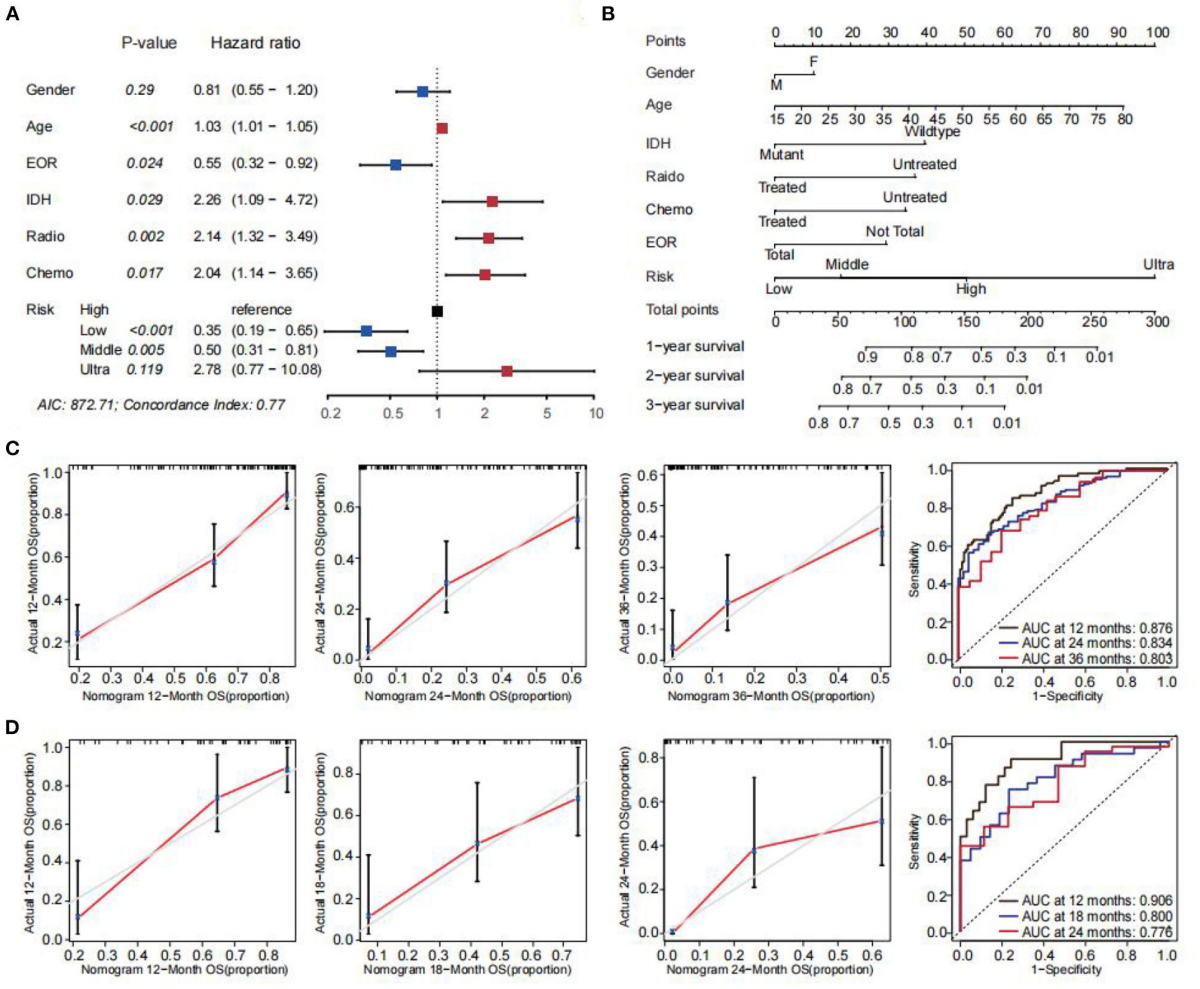
Construction of the prognostic model. **(A)** Forrest plot of the multivariate Cox regression analyses in the training set. *p* < 0.05 was considered statistically significant. **(B)** A nomogram constructed with age, sex, EOR, IDH mutation status, radiotherapy status, chemotherapy status, and risk score. For every patient, 3 lines were drawn upwards to validate the points received from the three nomogram predictors. The sum of these points is indicated on the total points' axis. A line was drawn downwards to assess the 12-, 24- and 36-month overall survival outcomes of GBM. **(C)** The calibration plot evaluating the nomogram. The Y-axis indicates actual survival outcomes, whereas the X-axis indicates the estimated 12-, 24-, and 36-month overall survival outcomes for training set patients. The ROC curves of the nomogram model in the training set. **(D)** Predicted 12-, 18-, and 24-month overall survival in the validation set. The ROC curves of the nomogram model in the validation set.

Patients with risk scores of 1 and 2 had slightly different OS. Additionally, patients with risk scores 3 and 4 had slightly different OS. A risk score 0 was defined as low risk; risk scores of 1 and 2 were defined as moderate risk; risk scores of 3 and 4 were defined as high risk; and a risk score of 5 was defined as ultrahigh risk. The baseline characteristics of the patients in the four groups are shown in Supplementary Material. Although sex was not significantly related to survival, this variable was retained in the multivariable models due to its clinical importance (Figure 1A). A nomogram was built using the training set to estimate the 12-, 24-, and 36-month survival probabilities (Figure 1B). This model showed good predictive ability with a global *p* value (log–rank) of 7.8321e−16, AIC of 872.71, and concordance index of 0.77. Calibration curves for the training set were established to predict 12-, 24-, and 36-month survival. Similarly, calibration curves for the validation set were established to predict 12-, 18- and 24-month survival since no patient in the validation set had an overall survival of greater than 36 months. The gray and red lines indicate the ideal survival rates and the observed survival rates, respectively. All the calibration curves were closely aligned with the 45-degree line, demonstrating good calibration. The scoring standard of the nomogram was obtained, and each patient's points were calculated. ROC curves were drawn, and the corresponding AUCs were calculated to compare the precision at 12 months (0.876), 24 months (0.834) and 36 months (0.803) in the training set. Moreover, the 12-, 18- and 24-month AUCs (0.906, 0.800, and 0.776, respectively) were calculated in the validation set. These findings validated the nomogram's good predictive ability (Figures 1C,D).

### Peripheral Blood Test-Related Genes

**Figure 2:**
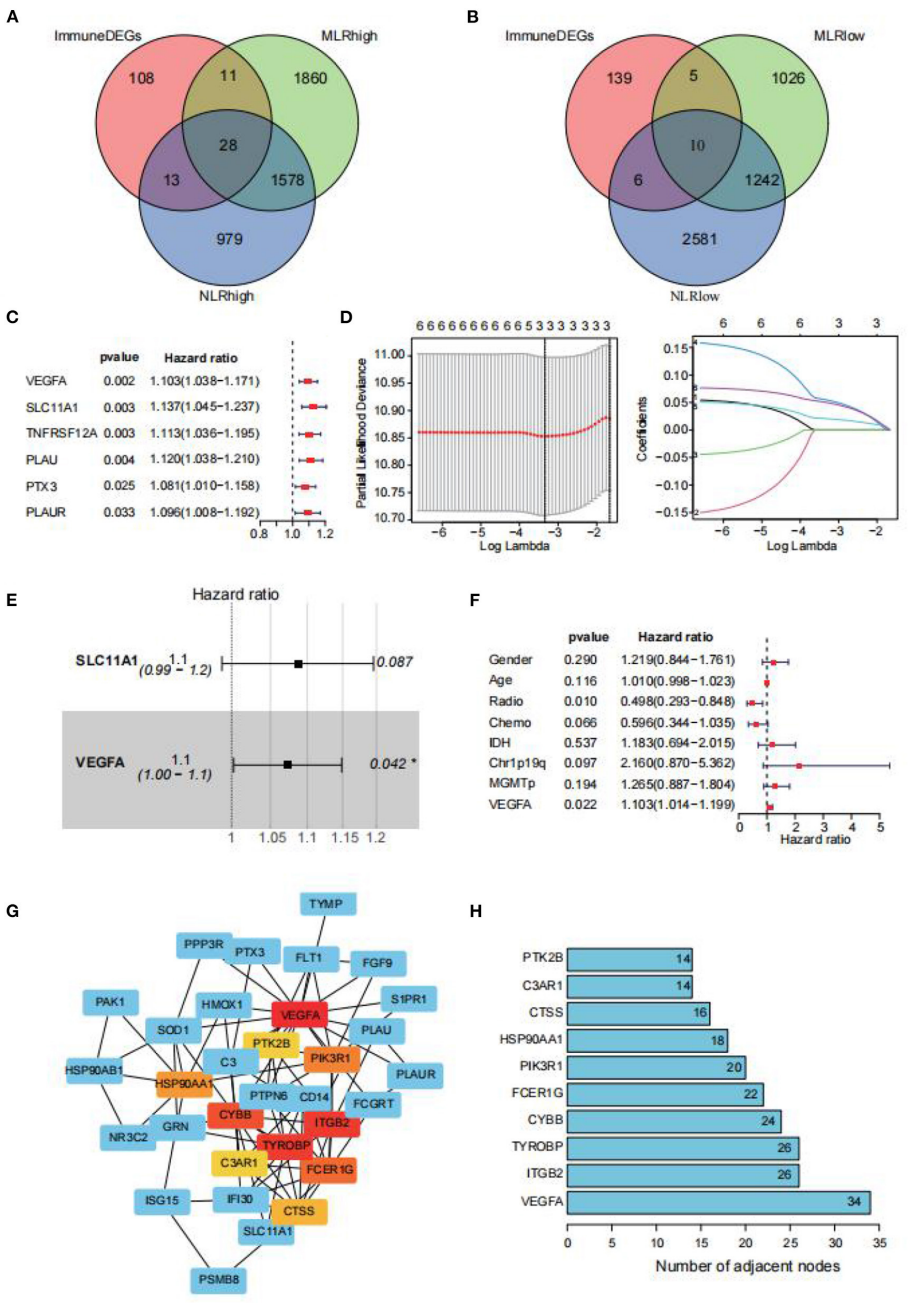
Venn diagrams of different gene sets. Venn diagrams show the crossed peripheral blood test-related genes between the NLR and MLR. **(A)** The intersection of upregulated genes. **(B)** The intersection of downregulated genes. Univariable Cox regression analysis, LASSO-penalized Cox regression analysis, and multivariable Cox analysis were implemented to select the key genes. **(C)** Univariate Cox regression showing the key genes (VEGFA, SLC11A1, TNFRSF12A, PLAU, PLAUR, and PTX3). **(D)** LASSO regression showing the significant key genes in the univariate Cox regression. **(E)** Multivariate Cox regression showing the final key gene (VEGFA, SLC11A1). **(F)** Multivariate Cox regression showing that VEGFA expression can be used as an independent indicator for the prognostic prediction of GBM patients. **(G)** PPI network of 38 genes. **(H)** Bar plot for the top 10 genes based on node numbers. VEGFA was top-ranked.

**Figure 3:**
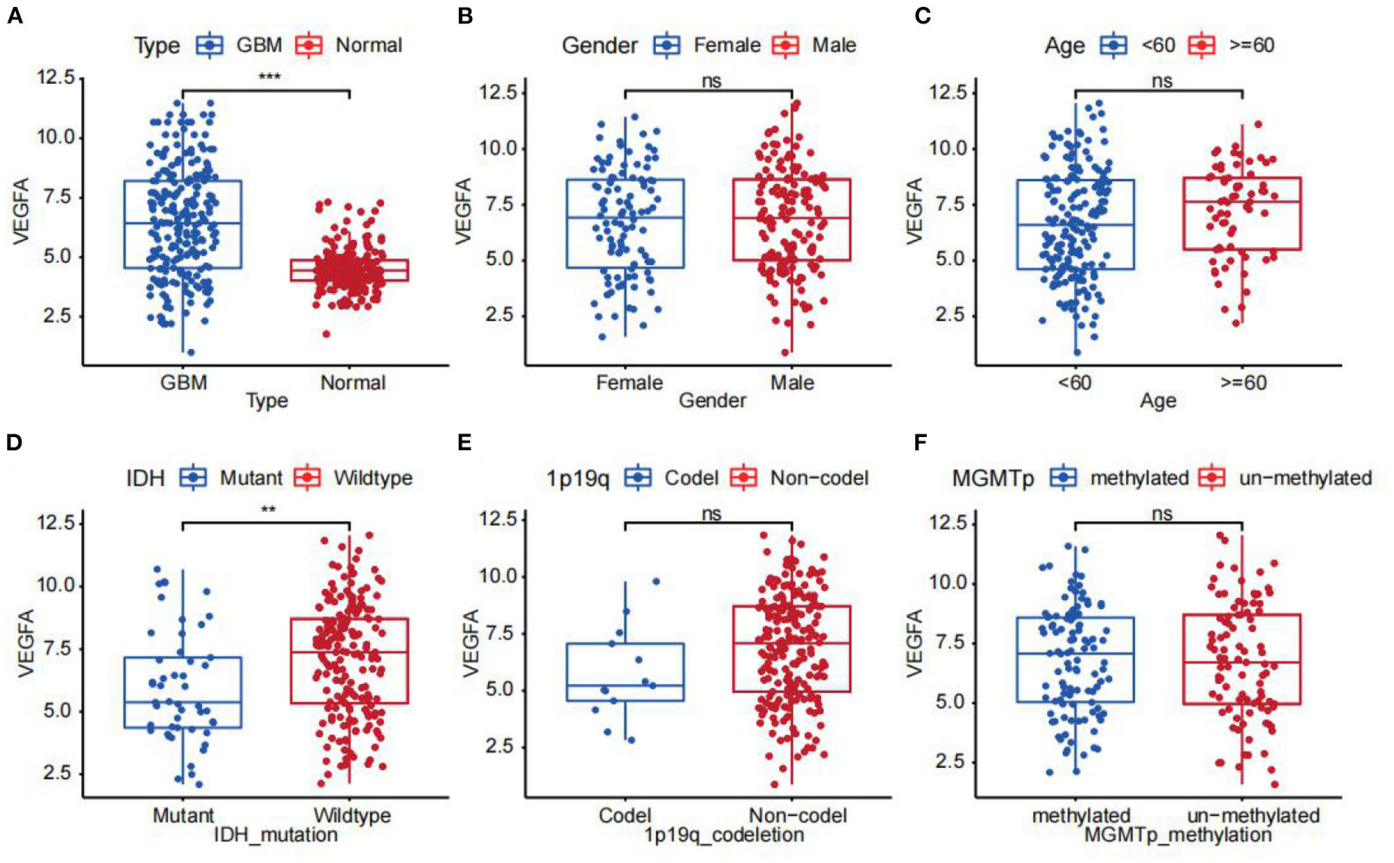
The correlation between mRNA expression of VEGFA vs. **(A)** type, **(B)** sex, **(C)** age, **(D)** IDH mutation status, **(E)** 1p19q codeletion status, and **(F)** MGMT promoter methylation status (**p < 0.01; ***p < 0.001).

NLR and LMR were associated with GBM patient prognosis. Nevertheless, the clinical significance of neutrophil, monocyte, and lymphocyte signatures from RNA sequencing data in GBM was not comprehensively evaluated. The final intersection included 38 genes (28 upregulated and 10 downregulated) (Figures 2A,B). Through univariate Cox regression analyses, six genes (VEGFA, SLC11A1, TNFRSF12A, PLAU, PTX3, and PLAUR) were significantly correlated with the OS of GBM patients in CGGA (Figure 2C). LASSO (Figure 2D) and multivariate Cox regression analyses further verified that two genes (SLC11A1 and VEGFA) represented the optimal combination for evaluating GBM patient prognosis (Figure 2E). SLC11A1 and VEGFA were more highly expressed in GBM samples compared with normal samples. The independent factors, including age, sex, radiotherapy status, 1p19q codeletion status, chemotherapy status, IDH mutation status, and MGMT promoter methylation status, were combined with the two genes. Only VEGFA (*p* = 0.022) was established to be an independent prognostic factor for GBM (Figure 2F). Patients with high VEGFA (HR = 1.103; CI = 1.014–1.199) expression had a significantly poorer prognosis. Cytoscape software was used to build a PPI network with 38 nodes and 94 edges based on the STRING database to identify the probable interaction network among the 38 genes (Figure 2G). The bar plot indicates the top 10 genes based on nodal numbers. These data reconfirmed that VEGFA was the core gene associated with high NLR and MLR (Figure 2H). This study also assessed the correlation between VEGFA and clinical factors. The results showed that VEGFA expression levels were markedly elevated in the IDH wild type compared with the IDH mutant type (Figure 3). Finally, the NLR and LMR of samples in CGGA, TCGA and GSE16011 were estimated by using CIBERSORT. The results showed NLR was statistically positively associated with expression of VEGFA in three datasets. However, the association between LMR and VEGFA were not statistically significant (Supplementary Figure 1).

### Multivariate Analysis of VEGFA

**Figure 4:**
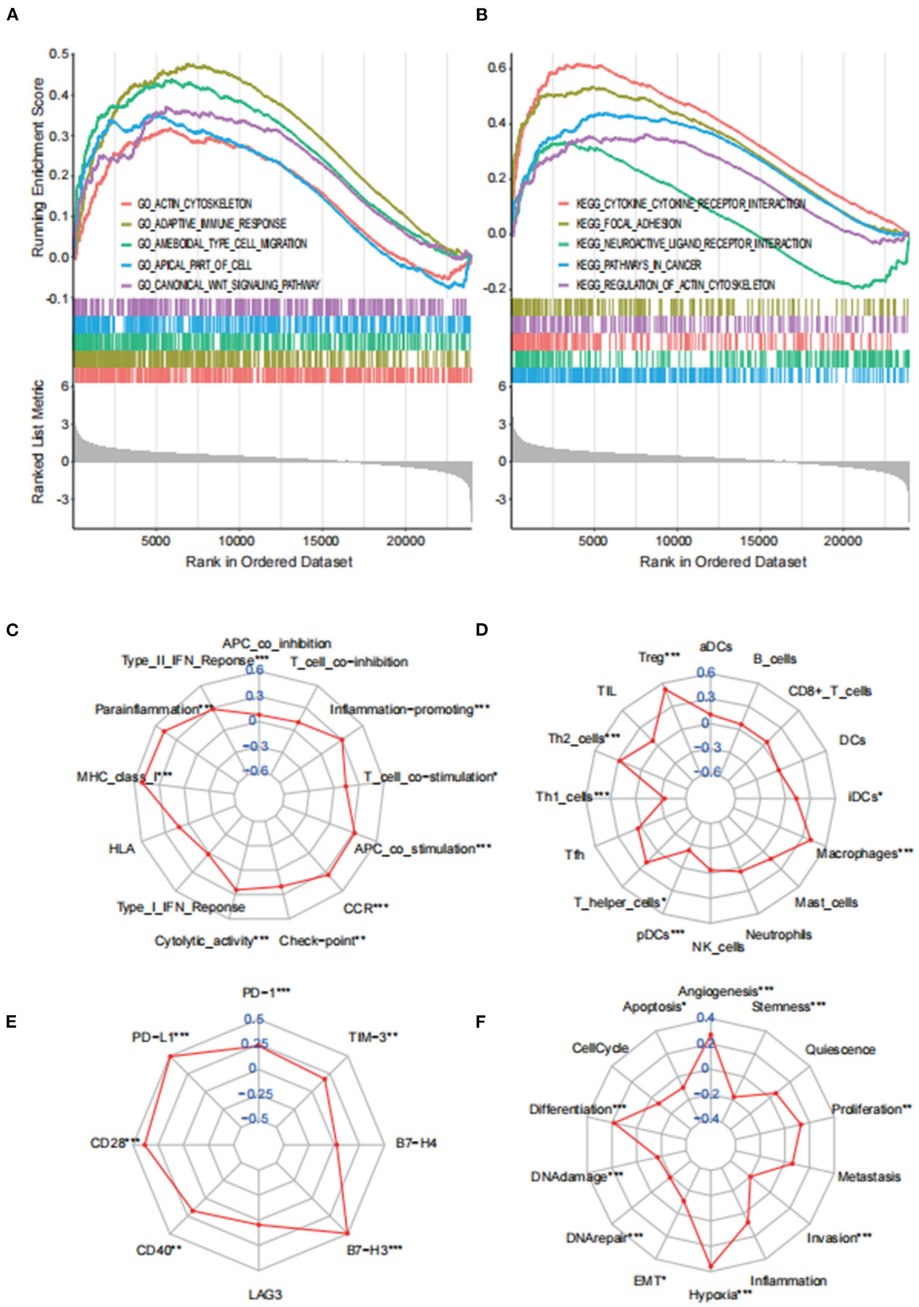
Analysis of VEGFA expression in GBM patients. **(A)** GO and **(B)** KEGG analyses via GSEA between the different VEGFA expression groups. **(C)** Radar plots showing the correlation between VEGFA expression and 16 immune-related cells, **(D)** 13 immune-related functions, **(E)** immune checkpoints in GBM, and **(F)** distinct functional states of cancer cells at single-cell resolution. (*p < 0.05; **p < 0.01; ***p < 0.001).

GSEA was applied to identify enriched features as well as functional differences between the low and high VEGFA expression groups using the top five entries of GO and KEGG terms. The high VEGFA expression group was found to be enriched in “ACTIN_CYTOSKELETON, ADAPTIVE_ IMMUNE_ RESPONSE, AMEBOIDAL_ TYPE_ CELL_ MIGRATION, APICAL_ PART_OF_ CELL and CANONICAL_ WNT_ SIGNALING_ PATHWAY” in GO and “CYTOKINE_ RECEPTOR_ INTERACTION, FOCAL_ ADHESION, NEUROACTIVE_ LIGAND_ RECEPTOR_ INTERACTION, PATHWAYS_ IN_CANCER and REGULATION_OF_ACTIN_ CYTOSKELETON” in KEGG. The enrichment scores of different immune cells and immune-associated functions as well as pathways were further evaluated based on the ssGSEA algorithm to assess the correlations between VEGFA expression and immune infiltration. Seven immune cells (Tregs, Th2 cells, Th1 cells, T helper cells, pDCs, iDCs, and macrophages) were markedly correlated with VEGFA expression. Moreover, nine immune-related functions (type II IFN response, parainflammation, CCR, MHC class I, inflammation promotion, checkpoint, T cell costimulation, APC costimulation, and cytolytic activity) were significantly correlated with VEGFA expression. In addition, six immune checkpoints (PD-1, PD-L1, TIM-3, B7-H3, CD40 and CD28) were closely associated with VEGFA, suggesting a potential association between VEGFA expression and immune checkpoint inhibitor treatment. ssGSEA confirmed that VEGFA levels might affect the immune status in the tumor microenvironment. Finally, single-cell RNA sequencing results showed that hypoxia increased VEGFA expression. The expression of VEGFA was significantly positively associated with hypoxia (R = 0.36) and angiogenesis (R = 0.27) (Figure 4).

## Discussion

Despite considerable advances in brain cancer therapy in the past decade, the prognosis of GBM is extremely poor (2). Comprehensive preoperative assessment tools are needed to classify GBM risk, thereby appropriately guiding further individualized treatment. Herein, peripheral blood test indicators and imaging indicators of 187 GBM patients were used to define a scoring system based on NLR, PLR, LMR, AFR, and CPVD. An individual survival nomogram for GBM patients was also developed and validated. The CPH survival model included basic patient information (age and sex), preoperative scoring system, extent of tumor resection, IDH status, and subsequent treatment after surgery. The AUC values of the ROC curve were 0.876 (12-month ROC), 0.834 (24-month ROC) and 0.803 (36-month ROC) in the training set and 0.906 (12-month ROC), 0.800 (18-month ROC) and 0.776 (24-month ROC) in the validation set. Compared to other models, our prediction models provide better prediction results and enhance the evaluation of the entire course of the disease. However, our risk score combined with multielement data included not only peripheral blood tests but also imaging factors and was finally combined with clinical risk factors to construct a more comprehensive and more individualized prognostic model in GBM. Many studies have constructed a series of risk score systems to evaluate the prognosis of gliomas. For example, Wu et al. constructed a risk score based on preoperative fibrinogen (F), the neutrophil to lymphocyte ratio (NLR), and albumin to globulin ratio (AGR) and found that a high F-NLR-AGR score was an independent predictor of poor prognosis; however, this study did not incorporate imaging analysis (27). Similarly, Zhang constructed a radiomics nomogram from 4,000 radiomics features, demonstrating a good predictive ability in both the training and validation sets without incorporating the peripheral blood analysis results (28). Finally, Wang et al. constructed a model based on radiomic signatures and blood urea nitrogen (BUN). However, our study used a larger sample size, and our model showed better predictive ability (29).

Recent studies have reported that GBM tumor cells may increase the neutrophil count both in the peripheral blood and around the tumor by secreting chemotactic factors, including G-CSF, VEGF, IL-1β, and IL-6, and neutrophils may promote tumor progression by secreting important cytokines, such as VEGF, IL-6, IL-8, elastases, and matrix metalloproteinases (30, 31). In addition, an increasing neutrophil count may regulate lymphocyte function by releasing reactive oxygen species and arginase, thereby inhibiting lymphocyte survival and normal cytotoxic function (32). It has also been reported that elevated platelet levels may promote tumor growth, angiogenesis, and dissemination by secreting crucial factors, such as VEGF (33). In addition, platelets release increased levels of soluble CD40, which is a known inhibitor of regulatory T cell recruitment that may promote the immunosuppressive microenvironment and eventually form an environment conducive to tumor growth (34). Abundant macrophage infiltration is a common feature of GBMs, but these tumor-associated macrophages (TAMs) lack apparent phagocytic activity in GBMs. Recent studies have shown that macrophages can be recruited and induced to become the M2 type by a wide variety of factors secreted by glioma cells, including IL-10, IL-4, IL-6, macrophage-colony stimulating factor (M-CSF), TGF-β, and prostaglandin E2 (PGE2), and promote cancer cell proliferation in GBMs (35, 36). In addition, M2-like macrophages also significantly promote angiogenesis and induce immunosuppression (37). Therefore, we found that neutrophil platelet count and macrophage count may be negatively correlated with lymphocyte count, and high NLR, PLR and MLR may promote angiogenesis in GBM. In our study, patients with a higher NLR (>4.16), higher PLR (>193.75) and lower LMR (≤2.52) had a poorer prognosis, and the outcomes were consistent with past studies.

Regarding the AFR, mounting evidence indicates that fibrinogen is an important regulator of tumor progression and systemic inflammatory responses in several malignant cancers and that hyperfibrinogenaemia is associated with a high invasiveness of GBM (38). Fibrinogen promotes tumor angiogenesis and increases the adhesive, migratory, and invasive abilities of tumor cells (39–41). Moreover, the physical barrier formed by platelet-fibrin deposition surrounding tumor cells can prevent NK cell destruction (42). Malignant tumor cells can synthesize fibrinogen, thus promoting tumor cell growth and angiogenesis through interactions with vascular endothelial growth factor and fibroblast growth factor-2 (43, 44). As a malnutrition and inflammation marker, a reduced albumin level is a risk factor for malignant tumors. Therefore, a lower AFR may imply inadequate antitumour immunity, malnutrition, and cancer-associated inflammation, which are unfavorable for cancer prognosis.

Matarredona et al. (20) indicated that the tumor location and the subventricular zone (SVZ) are related to a patient's prognosis. The SVZ of the adult human brain has several neural stem cells (NSCs), which can undergo multilineage differentiation. In the SVZ, NSCs are potential cells of origin containing driver mutations of human GBM (45). This study demonstrated that GBM patients with elevated CPVD have shorter OS than those with low CPVD. Thus, CPVD may be a marker for tumor invasion in GBM.

VEGFA is essential for physiological and pathological angiogenesis, and bevacizumab, a molecular-targeted drug, binds and neutralizes human VEGFA to suppress the VEGF signaling pathway. Given its antiangiogenic effects, bevacizumab has become the standard GBM treatment and positively affects the quality of life and survival of recurrent GBM patients. However, bevacizumab increases PFS but has no effect on OS. N. García-Romero et al. also reported that tumor growth of greater than 20% in GBM patients is independent of the VEGFA pathway and thus does not benefit from antiangiogenic therapy (46–48). Herein, differentially expressed genes between neutrophils and lymphocytes as well as monocytes and lymphocytes were screened by differential gene expression analysis. VEGFA was not only significantly related to survival but also ranked first in the protein–protein interaction (PPI) network map. Therefore, we confirmed that VEGFA expression was closely related to our risk score level, which was manifested through NLR, LMR and PLR. Based on the current achievements, we propose to perform prospective clinical trials in which we obtain VEGFA expression levels using ddPCR to detect and quantify ctDNA levels in GBM patient blood and cerebrospinal fluid to confirm the close correlation between VEGFA expression and peripheral blood tests. We also attempted to explore the prognostic predictive ability of the ctDNA level of VEGFA and whether ctDNA can be a potential molecular indicator of sensitivity or resistance to bevacizumab.

## Conclusions

In summary, the nomogram is a novel tool for predicting the prognosis of GBM patients, thereby informing individualized treatment. In addition, VEGFA may influence the immune microenvironment of GBM, leading to poor prognosis in GBM patients.

## Data Availability Statement

The original contributions presented in the study are included in the article/Supplementary Material, further inquiries can be directed to the corresponding author/s.

## Ethics Statement

The studies involving human participants were reviewed and approved by the Review Board of the First Affiliated Hospital of Wenzhou Medical University. The patients/participants provided their written informed consent to participate in this study.

## Author Contributions

CR, JJ, and QL conceived of and designed the study. JL and CW performed literature search. ZZ and ZW generated the figures and table. CR and MT analyzed the data. CR wrote the manuscript. QL critically reviewed the manuscript. ZS and QL supervised the research. All authors read and approved the final manuscript.

## Funding

This research was supported by grants from followings: Wenzhou Science and Technology Project under Grants, Y20190144, Key Research Project of Traditional Chinese Medicine of Zhejiang Province of China, 2019ZZ015, and Medical Health Science and Technology Research Project of Zhejiang Province of China, 2018KY515.

## Conflict of Interest

The authors declare that the research was conducted in the absence of any commercial or financial relationships that could be construed as a potential conflict of interest.

## Publisher's Note

All claims expressed in this article are solely those of the authors and do not necessarily represent those of their affiliated organizations, or those of the publisher, the editors and the reviewers. Any product that may be evaluated in this article, or claim that may be made by its manufacturer, is not guaranteed or endorsed by the publisher.

## Supplementary Material

Supplementary Table 1 Baseline characteristics of the patients in the four groups.

Supplementary Figure 1 Using RNA sequencing data from CGGA, TCGA and GSE16011 to estimate NLR and LMR by using CIBERSORT. The correlation between NLR and VEGFA was statistically significant, but the correlation between LMR and VEGFA had no statistically significant.
