# Supplementary material for: A Multielement Prognostic Nomogram Based on a Peripheral Blood Test, Conventional MRI and Clinical Factors for Glioblastoma
Source: Front Neurol. 2022 Feb 9;13:822735. doi: 10.3389/fneur.2022.822735 (PMC8893080; doi:10.3389/fneur.2022.822735)
Supplement: Supplementary Table 1 — Baseline characteristics of the patients in the four groups. [file Table_1.docx]

|  | Training set(N=131) | P-Value | Validation set(N=56) | P-Value |
| --- | --- | --- | --- | --- |
| Age(mean(SD))  Low  Middle  High  Ultra | 49.33(15.443)  54.15(14.533)  50.70(12.499)  57.50(0.707) | 0.406 | 59.62  55.00  62.14  57.50 | 0.416 |
| Gender(Male%)  Low  Middle  High  Ultra | 13/24(54.2)  43/75(57.3)  16/30(53.3)  1/2(50) | 0.956 | 7/13(53.8)  16/27(59.3)  9/14(64.3)  0/2(0) | 0.475 |
| IDH-Mutation(Mutant%)  Low  Middle  High  Ultra | 3/24(12.5)  7/75(9.3)  6/30(20.0)  0/2(0) | 0.464 | 0/13(0)  2/27(7.4)  0/14(0)  1/2(50.0) | 0.101 |
| EOR(Total-resection%)  Low  Middle  High  Ultra | 19/24(79.2)  61/75(81.3)  19/30(63.3)  2/2(100) | 0.230 | 12/13(92.3)  21/27(77.8)  13/14(92.9)  1/2(50.0) | 0.282 |
| Radiotherapy(treated%)  Low  Middle  High  Ultra | 18/24(75.0)  44/75(58.7)  19/30(63.3)  0/2(0) | 0.159 | 9/13(69.2)  16/27(59.3)  7/14(50.0)  0/2(0) | 0.325 |
| Chemtherapy  Low  Middle  High  Ultra | 21/24(87.5)  60/75(80.0)  17/30(56.7)  2/2(100) | 0.033 | 12/13(92.3)  24/27(88.9)  10/14(71.4)  1/2(50.0) | 0.196 |
