# Supplementary figures and images for: A Multielement Prognostic Nomogram Based on a Peripheral Blood Test, Conventional MRI and Clinical Factors for Glioblastoma
Source: Front Neurol. 2022 Feb 9;13:822735. doi: 10.3389/fneur.2022.822735 (PMC8893080; doi:10.3389/fneur.2022.822735)

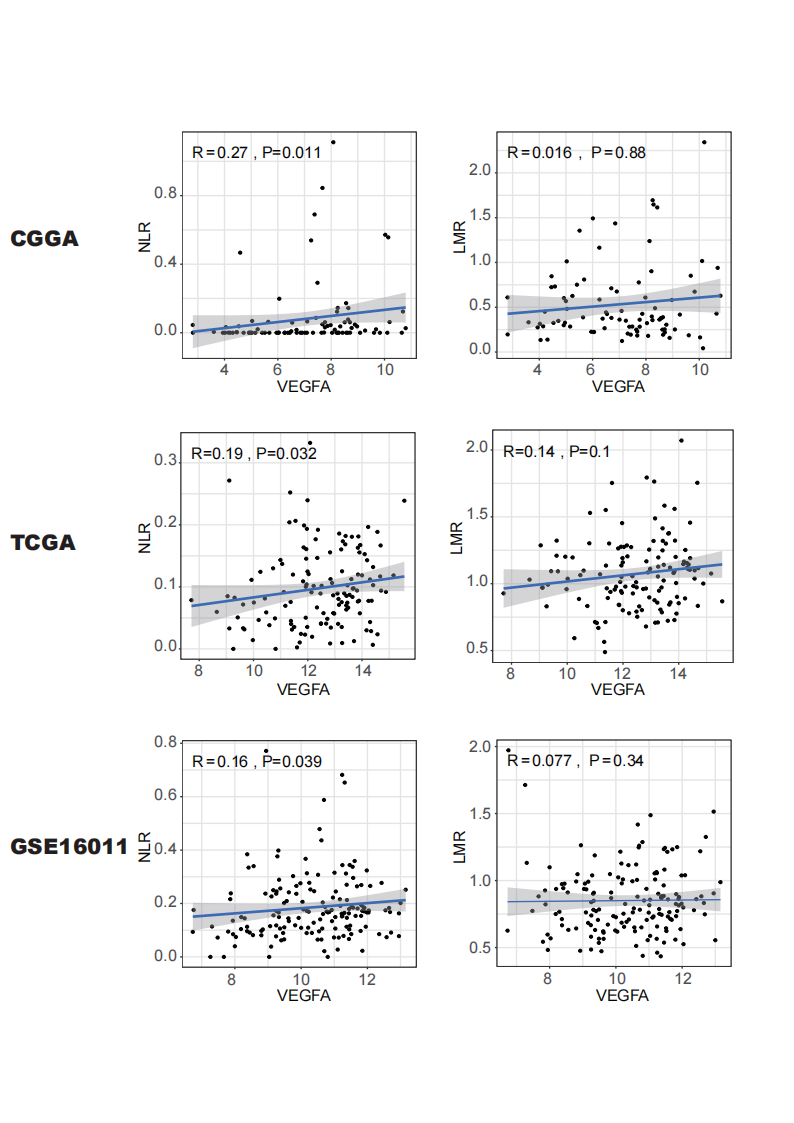

Supplement: Supplementary Figure 1 — Using RNA sequencing data from CGGA, TCGA and GSE16011 to estimate NLR and LMR by using CIBERSORT. The correlation between NLR and VEGFA was statistically significant, but the correlation between LMR and VEGFA had no statistically significant. [file Image_1.TIF]
